# Paraquat induced acute kidney injury and lung fibrosis: a case report from Bangladesh

**DOI:** 10.1186/s13104-018-3425-3

**Published:** 2018-05-30

**Authors:** Ishrat Tahsin Isha, Z. H. M. Nazmul Alam, Bidur Kanti Shaha, Md. Shafiqul Bari, Mohammad Zabed Jillul Bari, Fazle Rabbi Chowdhury

**Affiliations:** 1 Department of Medicine, Sylhet M.A.G Osmani Medical College Hospital, Medical College Road, Sylhet, 3100 Bangladesh; 2 Department of Medicine, Bangabandhu Sheikh Mujib Medical University, Dhaka, Bangladesh; 3 Centre for Tropical Medicine and Global Health, Nuffield Department of Medicine, University of Oxford, Oxford, UK

**Keywords:** Paraquat, Poisoning, Bangladesh, Acute kidney injury, Lung fibrosis

## Abstract

**Background:**

Since Bangladesh government issued a ban on the use of highly toxic WHO Class I pesticides, annual consumption of herbicides like Paraquat have been sharply increasing in the markets. Paraquat poisoning is an emerging public health threat and its high mortality rate is responsible for a significant number of deaths. Diagnostic limitations and unavailable sample at presentation have resulted in under-reporting and lack of awareness among the treating physicians, making Paraquat poisoning one of the most neglected toxicological emergencies. Herein, we present a case of Paraquat induced multi-organ failure and emphasis on pitfalls in the management.

**Case presentation:**

An 18-years-old healthy male was admitted in Sylhet M.A.G Osmani Medical College Hospital with history of attempted suicide by Paraquat ingestion. On admission, he had high serum creatinine but otherwise asymptomatic. He was discharged on day 10 when his renal functions returned to normal. But On day 15, he started having respiratory symptoms—unresponsive to any of the local treatments he received, and by day 30, he developed overt lung fibrosis. We present sequential blood picture, radiographs and CT scans demonstrating Paraquat induced kidney and lung injury over the course of 30 days.

**Conclusion:**

Paraquat poisoning can lead to death and fatal long-term consequences. All cases of Paraquat poisoning, regardless of symptoms, must be hospitalized and observed for early detection of complications. Distribution of Paraquat should be restricted and/or banned as 38 other countries have done so, which we believe will greatly reduce poisoning related mortality.

**Electronic supplementary material:**

The online version of this article (10.1186/s13104-018-3425-3) contains supplementary material, which is available to authorized users.

## Background

Since Bangladesh government issued a ban on WHO class I pesticides in 2000 [1], the use of herbicides have been increasing in our agriculture and most toxic herbicides like Paraquat entered into our market [2]. The annual consumption of Paraquat is sharply increasing in Bangladesh [2], so is the incidence of Paraquat poisoning, posing a major threat to public health. While the organophosphate (OP) poisonings still account for the majority of hospital admissions, the fatality of Paraquat poisoning cases has been an emerging concern.

Unfortunately, this compound has no effective antidote and rapidly causes multi-organ failure [3]. It has high mortality even with standard care and early management [3]. Diagnostic limitations and unavailable sample at presentation have resulted in under-reporting and lack of awareness among the treating physicians, making Paraquat poisoning one of the most neglected toxicological emergencies in Bangladesh. Herein this article, we present a case of Paraquat poisoning complicated by renal failure and lung fibrosis and emphasis on pitfalls in the management.

## Case presentation

**Fig. 1:**
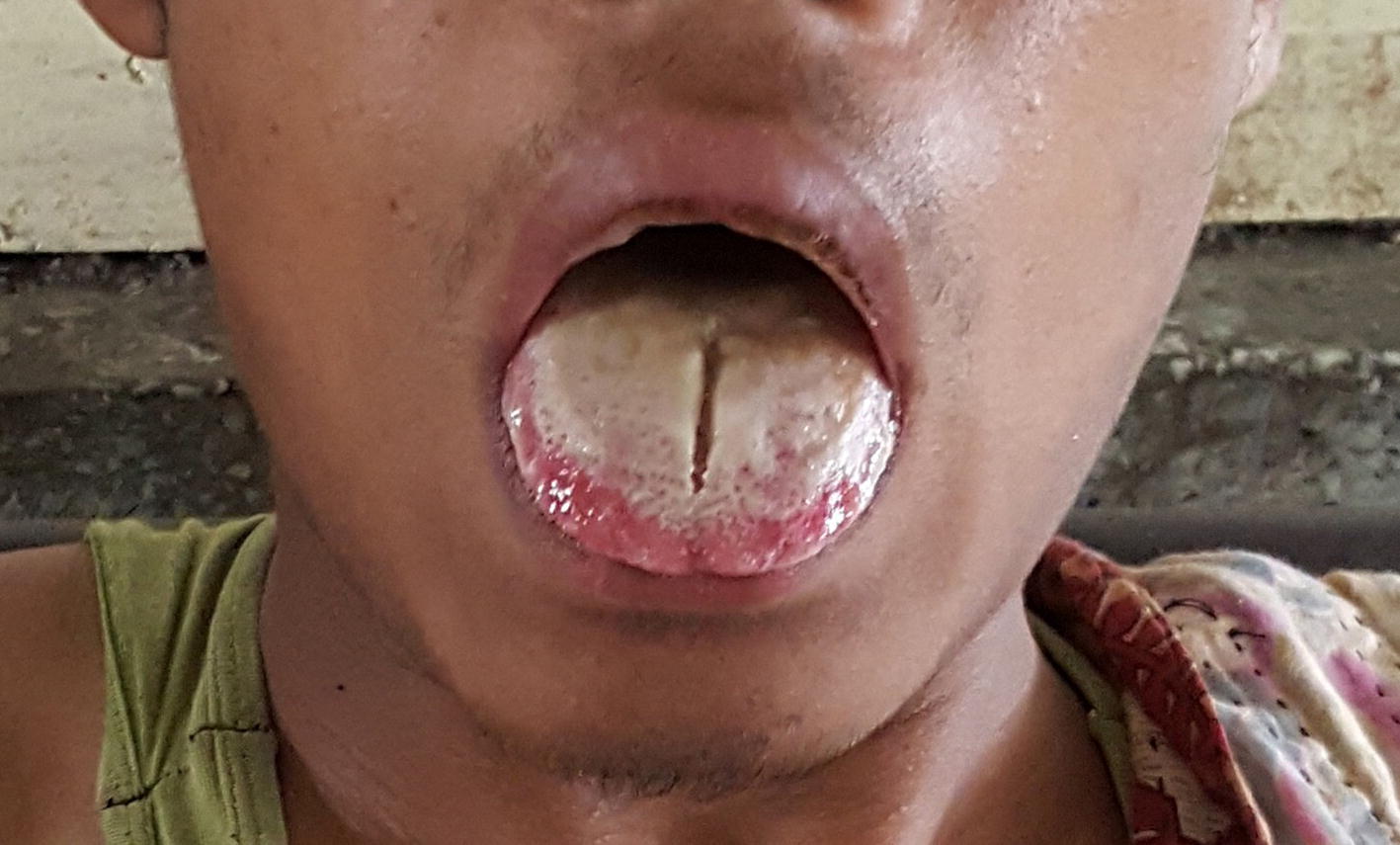
Paraquat tongue, within 24 h of ingestion

An 18-year-old healthy male was brought to the emergency room, Sylhet M.A.G Osmani Medical College Hospital with a history of attempted suicide by ingestion of about 30 ml of an unknown poison, later revealed to be Paraquat 20 SL. He was initially managed at a local health complex with gastric lavage, intravenous fluids, antiemetic, and H_2_ blocker, and referred to this tertiary hospital for further management. On admission, he had vomiting, difficulty in opening his mouth and inability to drink or swallow. He was conscious and oriented and had mucosal erosion of tongue (Fig. 1), palate, and lips with some mucosal bleeding having poisoning severity score (PSS) grade one. His heart rate was 78/min and regular, blood pressure was 100/60 mm Hg, respiratory rate was 20/min and temperature 98°K. Pupils were normal and reacting to light. Oxygen saturation was 98% on room air. Both lung fields were clear on auscultation. Other systemic examinations were normal.

**Fig. 2:**
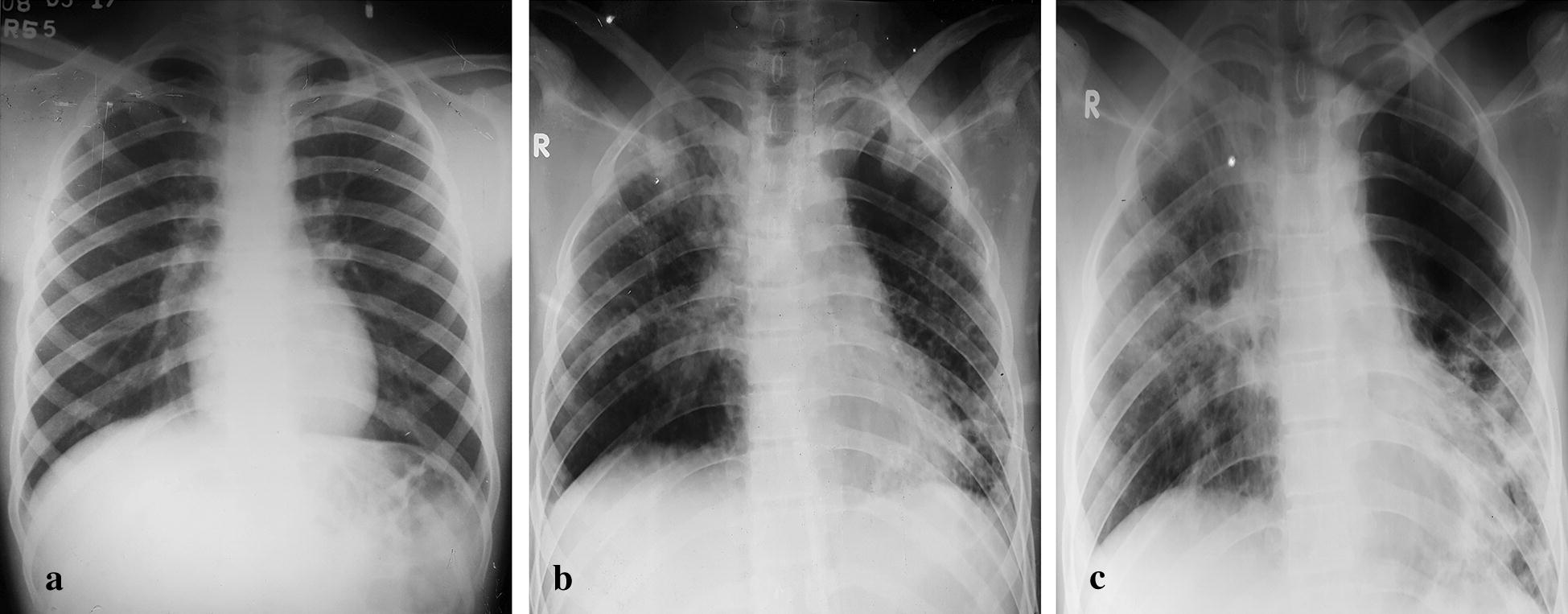
Serial chest radiographs after Paraquat poisoning. **a** Day 1—Normal. **b** Day 15—Diffuse alveolar shadowing predominantly involving left mid and lower zone. **c** Day 30—Diffuse alveolar shadowing extending to right apical and mid zone

Laboratory investigations revealed high serum creatinine (PSS grade 2). Complete blood count, serum ALT and electrolytes were within normal limits (Table 1). Chest radiograph was also normal (Fig. 2a).

**Table 1:** Laboratory profile after Paraquat poisoning

Investigation	Day 1	Day 10	Day 30
Haemoglobin	13.6	11.5	13.7
WBC	10,600	7200	8100
Platelets	276,000	379,000	332,000
Serum creatinine	4.32	2.1	0.84
Bilirubin total	2.0	–	–
ALT	36	13.7	48
Na^+^/K^+^	138.9/3.71	136.7/4.12	132.3/4.71
HCO_3_^−^	25.8	–	–
SpO_2_	98%	–	97%
Others	–	–	Sputum for AFB: negative

To rule out OP poisoning, atropine challenge test was performed which was negative. He was managed conservatively and was soon able to swallow liquids. He was discharged on day 10 when his renal function settled (Table 1).

**Fig. 3:**
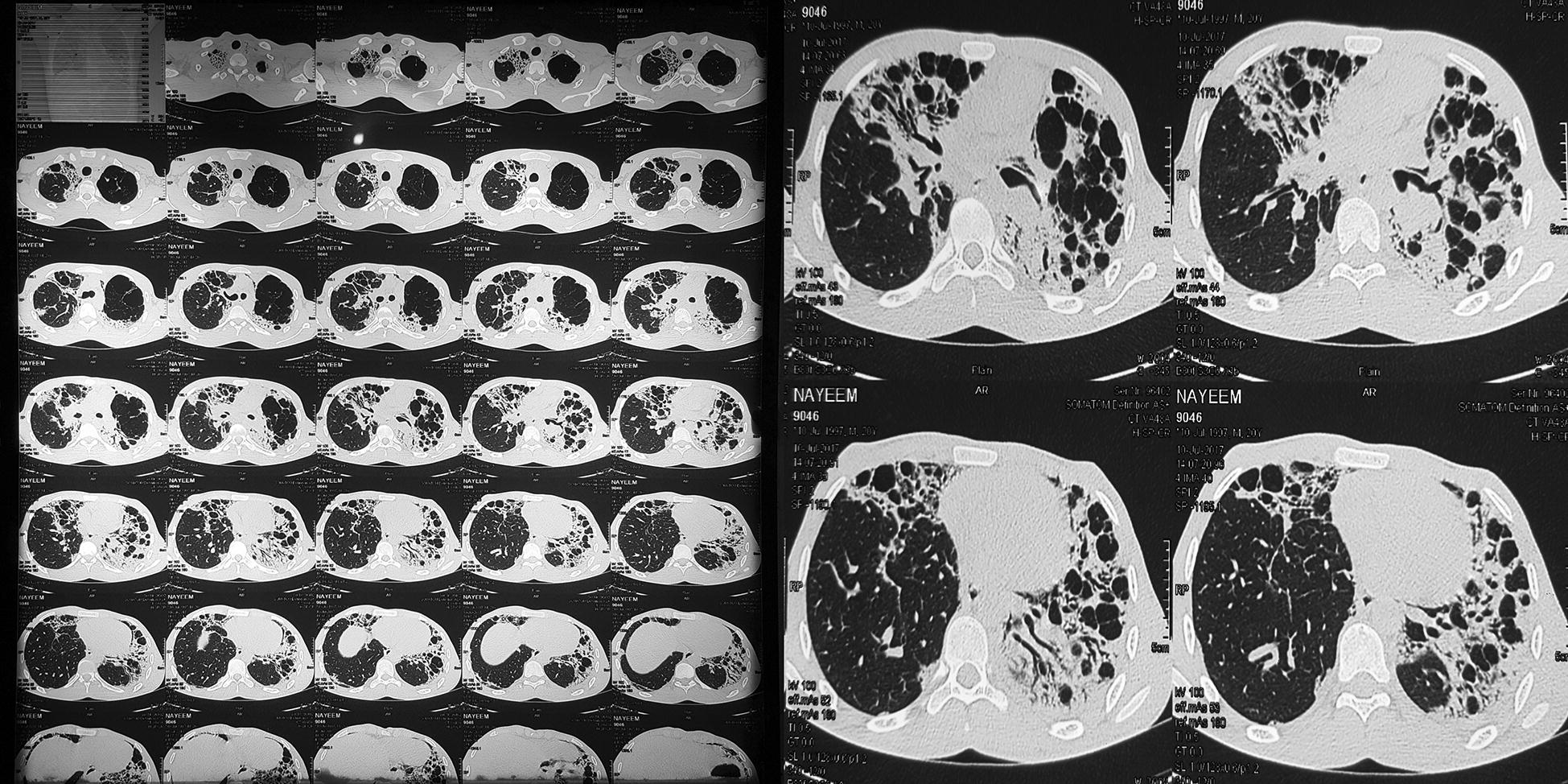
High resolution CT scan of chest demonstrating bilateral pulmonary fibrosis 30 days after Paraquat poisoning

On day 15, he developed irregular fever, shortness of breath and non-productive cough and as these symptoms progressed, he consulted with a local physician. Chest radiograph revealed diffuse consolidation (Fig. 2b) and he was prescribed a 14-day course of antibiotics. But his condition deteriorated and he had to get admitted to the hospital on day 30. The lesions on his chest radiograph showed bilateral diffuse alveolar shadowing (Fig. 2c) and a high-resolution CT scan of chest was obtained, which revealed bilateral pulmonary fibrosis (Fig. 3).

He was started on cyclophosphamide and methylprednisolone and his condition was static. There was no further deterioration during his stay. (Additional file 1)

## Discussion and conclusion

Paraquat (1,1′-Dimethyl-4,4′-bipyridinium dichloride) has the ability to generate highly reactive oxygen and nitrite species which cause cellular damage and apoptosis in many organs [3]. The clinical manifestations depend upon the quantity ingested. Ingestion of large amounts of liquid concentrate (> 50–100 ml of 20% w/v) results in fulminant multi-organ failure and death within several hours to a few days [3]. Ingestion of smaller quantities usually leads to toxicity to two key target organs, kidneys and lungs, developing over days to weeks [3, 4].

An ingestion of 10–15 ml of 20% w/v Paraquat solution is considered lethal [4]. The estimated amount taken by our patient was 30 ml of 20 SL solution, which was quite high. Following ingestion, the herbicide induces a burning sensation of the mouth and throat, gastrointestinal irritation, abdominal pain, nausea, vomiting, and diarrhea [3]. In our patient, all the initial symptoms were due to erosion and irritation. Activated charcoal or Fuller’s earth was not used due to unavailability.

Irrespective of its route of administration, it is rapidly distributed in most tissues, with the highest concentration found in the lungs and kidneys [3, 4]. It is actively taken up by the type II pneumocytes against a concentration gradient. Lung damage occurs in two phases, initially from destructive alveolitis over  one to three days followed by proliferative phase leading to fibrosis [3, 4]. Excretion of Paraquat is biphasic, owing to lung accumulation and occurs largely in the urine [4, 5]. In our patient, renal failure was evident by a rise of serum creatinine to 4.32 mg/dl on day 1, which subsequently normalized by day 30 with conservative treatment. Respiratory symptoms appeared at the end of 2nd week and features of lung fibrosis became evident within 1 month, consistent with hallmark lung findings of Paraquat poisoning [6].

This case posed a number of diagnostic challenges to the medical team. Paraquat poisoning is an emerging problem of Bangladesh and never been reported from this region [7]. Therefore, there was considerable confusion regarding the identity of the pesticide ingested. This patient got gastric lavage (which made his condition worse) and test doses of atropine initially and might have looked upon as OP poisoning. Later he was confirmed as a case of Paraquat poisoning after examining the container. Secondly, the initial clinical features were nonspecific. Initial symptoms of vomiting and mucosal ulceration mislead to other corrosive agents. Thirdly, failure to anticipate the complications led to an early discharge of the patient. Subsequently, he developed overt lung fibrosis within 30 days. Methylprednisolone and cyclophosphamide therapy was not given initially (started later), which was a major pitfall in the management of our patient. Early initiation of these therapies might reduce the accumulation into lung [3].

Up to date, 38 countries have issued a ban on Paraquat including the European Union, Sri Lanka, Vietnam and South Korea [8–11]. Bans of W.H.O class I pesticides is proved to significantly lower the suicide rates in Bangladesh [1]. Therefore it is the high time to implement such regulation on Paraquat also.

Paraquat poisoning can lead to death and fatal long-term consequences. Unfortunately, there is no available antidote, which makes it more hazardous. All cases, regardless of symptoms, must be hospitalized and observed for early detection of complications. We recommend the government should look into the problem at large and issue a ban on Paraquat which will effectively lower the poisoning death-rates.

## Additional files

**Additional file 1.** Timeline of clinical events.

## Abbreviations

PSS: poisoning severity score
AFB: acid fast bacilli
ALT: alanine transaminase
OP: organophosphate

## References

[1] Chowdhury FR, Dewan G, Verma VR, Knipe DW, Isha IT, Faiz MA (2018). Bans of WHO class I pesticides in Bangladesh—suicide prevention without hampering agricultural output. Int J Epidemiol.

[2] Pesticides use. http://www.fao.org/faostat/en/#data/RP. Accessed 15 Apr 2018.

[3] Gawarammana IB, Buckley NA (2011). Medical management of paraquat ingestion. Br J Clin Pharmacol.

[4] Wunnapuk K, Mohammed F, Gawarammana I, Liu X, Verbeeck RK, Buckley NA (2014). Prediction of paraquat exposure and toxicity in clinically ill poisoned patients: a model based approach. Br J Clin Pharmacol.

[5] Houze P, Baud FJ, Mouy R, Bismuth C, Bourdon R, Scherrmann JM (1990). Toxicokinetics of paraquat in humans. Hum Exp Toxicol.

[6] Lee SH, Lee KS, Ahn JM, Kim SH, Hong SY (1995). Paraquat poisoning of the lung: thin-section CT findings. Radiology.

[7] Bari MS, Chakraborty SR, Alam MMJ, Qayyum JA, Hassan N, Chowdhury FR (2014). Four-year study on acute poisoning cases admitted to a tertiary hospital in Bangladesh: emerging trend of poisoning in commuters. Asia Pac J Med Toxicol.

[8] EU Court Reimposes Ban on Paraquat Weed killer. http://www.reuters.com/article/environment-eu-paraquat-dc/eu-court-reimposes-ban-on-paraquat-weedkiller-idUSL1166680020070711. Accessed 12 Oct 2017.

[9] Pearson M, Zwi AB, Buckley NA, Manuweera G, Fernando R, Dawson AH (2015). Policymaking ‘under the radar’: a case study of pesticide regulation to prevent intentional poisoning in Sri Lanka. Health Policy Plan.

[10] Cha ES, Chang SS, Gunnell D, Eddleston M, Khang Y-H, Lee WJ (2016). Impact of paraquat regulation on suicide in South Korea. Int J Epidemiol.

[11] PAN Vietnam Welcomes the Ban of Paraquat and 2,4-d. http://panap.net/2017/02/pan-vietnam-welcomes-the-ban-of-paraquat-and-24-d/. Accessed 12 Oct 2017.

