# Supplementary material for: Paraquat induced acute kidney injury and lung fibrosis: a case report from Bangladesh
Source: BMC Res Notes. 2018 May 30;11:344. doi: 10.1186/s13104-018-3425-3 (PMC5975581; doi:10.1186/s13104-018-3425-3)
Supplement: Supplementary file 1 — Additional file 1. Timeline of clinical events. [file 13104_2018_3425_MOESM1_ESM.docx]

Timeline of events

May 5, 2017: Patient ingested Paraquat

May 14, 2017: Discharged after renal function improved

May 19, 2017: Consulted local physician with SOB, dry cough. Started on a 14-day course of oral Clarithromycin

June 3, 2017: Again admitted, diagnosed with lung fibrosis

June 5, 2017: Started on injectable Methylprednisolone and oral Cyclophosphamide

June 28, 2017: Discharged with advice to follow-up monthly

July 30, 2017: Condition was static on follow-up
